# Exploring Affordable Curative Therapy for Sickle Cell Disease in Africa: A Comprehensive Overview

**DOI:** 10.1002/ajh.70221

**Published:** 2026-03-14

**Authors:** Adetola A. Kassim, Alexis A. Thompson, Punam Malik

**Affiliations:** ^1^ Department of Medicine, Division of Hematology and Oncology Vanderbilt‐Meharry Center for Excellence in Sickle Cell Disease, Vanderbilt University Medical Center Nashville Tennessee USA; ^2^ Department of Pediatrics, Cancer and Blood Disease Institute Cincinnati Children's Hospital Medical Center Cincinnati Ohio USA; ^3^ Department of Pediatrics, Children's Hospital of Philadelphia University of Pennsylvania Perelman School of Medicine Philadelphia Pennsylvania USA

**Keywords:** allogeneic hematopoietic stem cell transplant, gene therapy, low‐middle‐income countries, sickle cell disease

## Abstract

The practical aspects of developing curative treatments for sickle cell disease (SCD) in Africa, such as gene therapy and hematopoietic stem cell transplantation, involve strengthening healthcare infrastructure, training healthcare professionals, establishing regional treatment centers, and creating national SCD programs. The costs associated with gene therapy and stem cell transplants can be prohibitive, especially in low‐ and middle‐income countries. Strategies to address affordability, including local manufacturing, government funding, and partnerships with global health agencies, are essential to ensure equitable access. Establishing ethical and regulatory guidelines while raising awareness among patients and communities is critical. Early diagnosis through newborn screening and adequate clinical care programs are vital for identifying individuals with SCD who could benefit from curative therapies and other interventions. Addressing cultural beliefs and promoting positive attitudes toward SCD and its management is essential. While curative therapies offer hope, hydroxyurea and other disease‐modifying therapies with established clinical benefits are more accessible in many settings. Prioritizing these medications ensures that patients with SCD are medically prepared to receive curative therapies while simultaneously building capacity for advanced treatments, thus creating a pragmatic approach. Lastly, international collaboration and partnerships among researchers, global health agencies, and local organizations are vital for sharing knowledge, resources, and best practices in SCD management.

## 1 The Case for Curative Therapy for Sickle Cell Disease in Sub‐Saharan Africa

Sickle cell disease (SCD) is one of the most common genetic diseases worldwide. It is estimated that about 500, 000 babies are born each year, mainly in sub‐Saharan Africa, with nearly 8 million people living with the disease globally, posing a major public health challenge [1]. SCD is an autosomal recessive condition caused by a point mutation in Codon 6 of the β‐globin gene, resulting in an amino acid change from glutamic acid to valine (p.Glu6Val in the *HBB* gene). This mutation results in the production of sickle hemoglobin (HbS) instead of the normal hemoglobin A (HbA). Individuals who are homozygous for the HbS allele (HbSS) and those with HbSβ^0^‐thalassemia represent the most common and severe form of SCD, known as sickle cell anemia (SCA), which accounts for about 60% of cases. The remaining cases include compound heterozygosity for HbS and hemoglobin C, known as HbSC (30% of SCD patients). Coinheritance with other genes that encode β‐globin chain variants, such as D^‐Punjab^, E, and O^‐Arab^, represents much rarer forms of SCD seen worldwide with varied clinical manifestations [2, 3]. Survival in Africa is reported to be as low as 10%, with the highest mortality rate occurring in children under the age of 5 [4, 5]. The gradual introduction of interventions that have improved childhood survival in high‐income countries (HIC), such as newborn screening, enrollment in comprehensive care programs, infection prevention, and prompt diagnosis and treatment of acute life‐threatening events, is likely to enhance childhood survival to around 50%–90% in low‐middle‐income countries (LMIC) [6, 7].

Unfortunately, despite progress for children with SCD over the last 50 years in HICs, the life expectancy for adults with SCD has not improved significantly [8]. Improvements in supportive care have shifted the primary cause of death among SCD patients from infections to cardiopulmonary disease [9]. SCD negatively impacts the quality of life and reduces life expectancy for those affected [10]. Physical complications include painful vaso‐occlusive crises (VOC), stroke, acute chest syndrome, splenic sequestration, long‐term pulmonary and renal issues, and avascular necrosis of the joints [2]. Children and adolescents face not only the physical stress of the illness but also the psychological stress, including the chronic nature of complications, limitations in educational and recreational activities due to symptoms, and financial burdens on their families [11]. Such childhood chronic illness can lead to poor psychological adjustment, behavioral problems, somatic complaints, lower levels of school and social competence, anxiety, and depression [12].

Recent FDA approval of new disease‐modifying therapies (i‐glutamine, crizanlizumab, and voxelotor), along with hydroxyurea therapy [13], has not impacted this age‐related chronic organ dysfunction, especially affecting the CNS, heart, lungs, and kidneys [14, 15]. Comorbidity has been independently linked to lower survival rates, and an additive effect was observed, indicating that patients with more adverse endpoints experienced worse outcomes. Table 1 reviews the predictors of decreased survival in patients with SCD [16, 17, 18, 19, 20, 21, 22, 23, 24]. Among sickle cell genotypes, survival curves indicated that HbSS and Sβ^0^ predicted the poorest outcomes, followed by HbSC and Sβ^+^ [2]. Stroke is the leading cause of neurologic morbidity in children [22, 23, 25]. The incidence of overt stroke remains approximately 11% in most LMIC due to the lack of routine use of transcranial Doppler (TCD) to identify children with SCD at high risk of stroke, along with regular blood transfusions [22, 23]. Silent cerebral infarcts (SCIs) are the most commonly recognized cause of neurologic injury in patients with SCA, identified in more than 20% of children and 50% of adult patients [25]. SCIs are linked to an average decline of 5 full‐scale IQ points, resulting in poor academic performance and future overt issues with strokes [26, 27]. In an LMIC like Nigeria, where roughly 50% of all infants with SCA worldwide are born, resulting in about 150, 000 total births annually [28], and with a stroke incidence of 88 strokes per 100 person‐years among children, this results in a stroke burden of 16, 500 cases each year in children, along with SCIs and their sequelae. Although hydroxyurea and chronic blood transfusion therapy are employed for both primary and secondary stroke prevention, they are regarded as palliative strategies and are also not readily available in LMIC [29, 30, 31, 32, 33, 34]. Patients with SCD almost always develop sickle cardiomyopathy, which involves progressive cardiac fibrosis, diastolic dysfunction, increased pulmonary venous pressure, and a 20%–30% risk of sudden death from cardiac arrhythmias unless early disease‐modifying treatments are started [35, 36, 37, 38, 39].

**TABLE 1:** Identified factors linked to lower survival in SCD patients.

Characteristic	Hazard ratio	95% Confidence interval [CI]	*p*	Comment	References
Pulmonary hypertension^a^	2.34	1.32, 4.14	0.0036	25% positive predictive value by echo; right heart catheterization was 6%–10%	[16, 17, 18, 19]
Low forced expiratory volume in 1 s (FEV_1_)	1.07	1.00–1.04	0.037	Based on spirometry measures at > 21 years of age, based on ATS guidelines	[20]
Higher acute chest syndrome incidence rate	1.27	0.81, 1.99	< 0.2945	Hazard ratio per event/year ~10	[15, 16, 21]
ESRD	2.80	2.31, 3.38	—	Attenuated by pre‐dialysis nephrology care	[15, 16, 21]
Proteinuria	1.86	1.27, 2.73	0.0014	≥ 1 + proteinuria by dipstick	[16]
Cerebrovascular event (CVE)^b^	1.95	1.34, 2.83	0.0005	Defined as at least one stroke, seizure, or transient ischemic attack (TIA)	[16]
Stroke (CVA)	1.64	1.04, 2.58	0.0335	The prevalence of CVA (4.01%) and incidence (0.61 per 100 patient‐years) are observed in HbSS patients, but CVA occurs in all common genotypes	[16, 22, 23]
Seizures	2.54	1.62, 3.98	< 0.0001	Poor prognostic factor	[16]
sVCAM‐1 levels	2.032	n/a	0.0003	Related to underlying vascular damage and inflammation	[16]
Leg ulcer	1.66	1.12, 2.47	0.0118	Predictor of early mortality	[16]
Vaso‐occlusive pain episodes (VOE)^c^	1.74	1.18, 2.58	0.0054	Poorer survival with VOE > 4×/year	[16]
Chronic opiate use	1.68	1.08, 2.61	0.0204	Long OR short acting narcotics (daily)	[13]
Antihypertensives^d^	1.74	1.10, 2.73	0.0168	Poor prognostic factor	[16]
Clustering of end‐organ disease	4.20	1.63, 10.82	0.003	Multiple‐organ impairment (affecting > 1 organ) Organ System Severity Score (those with scores of > 1; HR = 1.502, *p* = 0.0263) [13]	[14, 16]

^a^ Defined as tricuspid regurgitant velocity ≥ 2.5 m/s by echocardiography; pro‐BNP levels, 6‐min walk tests (6MWTs) and right heart catheterization (RHC).

^b^ Cerebrovascular event (CVE)—defined as at least one stroke, seizure, or transient ischemic attack (TIA), was associated with decreased survival.

^c^ Defined as pain crisis requiring inpatient admission within 1 year.

^d^ Relationship of antihypertensive medication use to survival was not significant when analyzed with GFR as a covariate.

The overall burden of illness and death among people with SCD is high, and limited resources in LMICs cannot cover the costs of healthcare for monitoring, preventing, and treating these age‐related complications. Curative therapies provide the most practical way to prevent a lifetime of SCD suffering and to lower healthcare costs. However, they must be accessible and affordable in LMICs, where most SCD patients reside. In this review, we examine current advances in curative therapies and outline a plan to making these treatments applicable in LMICs.

## 2 Allogeneic Hematopoietic Stem Cell Transplantation as a Potential Curative Treatment for SCD

### 2.1 Background

Allogeneic hematopoietic cell transplantation (alloHCT) is becoming an increasingly accessible curative treatment for SCD. It is currently recommended for individuals with severe SCD and complications such as stroke, acute chest syndrome, recurrent pain crises, nephropathy, retinopathy, osteonecrosis in multiple joints, and priapism [40, 41, 42, 43]. Compared to other disease‐modifying therapies like regular transfusions and hydroxyurea, alloHCT can not only stop ongoing vaso‐occlusive crises and slow the progression of organ damage but also potentially reverse some existing organ damage [44]. Reducing or preventing further organ damage is a key goal for curative therapies for SCD, with current evidence indicating that the primary cause of death is cardiopulmonary disease [9]. Mixed donor chimerism after alloHCT for SCD can lead to the resolution of disease symptoms but requires stable donor chimerism greater than 25% [45]. Low‐intensity regimens depend on tolerance induction and the establishment of mixed‐donor chimerism. However, recent evidence indicates that the rate of hematologic malignancy is significantly higher following alloHCT for SCD compared to SCD patients who have not undergone transplantation [46]. The persistence of host cells exposed to low‐dose radiation, which can trigger myeloid malignancies, has been implicated. Pre‐existing myeloid mutations and prior inflammation may predispose patients to malignancy [47]. Therefore, the current goal of alloHCT for SCD is to achieve complete donor chimerism.

Historically, challenges to the universal acceptance of alloHCT for SCD have included the availability of suitable donors, especially human leukocyte antigen (HLA)–matched related donors, the toxicity of the conditioning regimen, and the risk of graft‐versus‐host disease (GVHD). Individuals with SCD also face an increased risk of complications during stem cell transplantation due to pre‐existing SCD‐related tissue damage, endothelial activation, and inflammation. This highlights the importance of careful recipient and donor selection, optimal conditioning intensity, and effective GVHD prevention, as well as the need to develop specific endpoints for Patient‐Reported Outcomes (PRO) in SCD. Event‐free survival (EFS) in most published studies of alloHCT for SCD is defined as the time from transplantation to the occurrence of graft rejection or death from any cause. Secondary objectives typically includes estimates of overall survival (OS), engraftment rate, chimerism levels, and the incidence of acute and chronic GVHD. Regimen‐related toxicity is usually defined as Grade 4 or 5 in any organ system or Grade 3 in the pulmonary, cardiac, renal, central nervous system (including PRES), TMA, oral, or mucosal systems [48]. Health‐related quality of life (HRQL) is assessed using validated questionnaires, such as the Child Health Questionnaire (CHQ) and the PROMIS (Patient‐Reported Outcomes Measurement Information System), which evaluate and monitor physical, mental, and social health in adults and children before and after transplant [49, 50, 51]. Currently, there are no randomized clinical trials comparing alloHCT with conservative approaches in patients with SCD.

Current alloHCT approaches are addressing these unmet needs, ushering in an era marked by increased donor availability, reduced toxicity, and a decline in GVHD. A multidisciplinary guideline panel formed by the American Society of Hematology (ASH) produced eight recommendations with very low certainty of evidence, primarily focusing on which patients should be considered for alloHCT [40]. Addressing these numerous factors is essential for alloHCT to serve as a curative therapy option in sub‐Saharan Africa. To achieve this, selecting an approach that is feasible, practical, and cost‐effective is essential, aiming to minimize morbidity and mortality related to the procedure and improve patient outcomes.

### 2.2 Current Approaches for AlloHCT as a Curative Option for SCD

The era of alloHCT as a potential cure for all eligible individuals with SCD is now. Myeloablative HLA‐matched sibling donor transplants are an established curative approach for children and adolescents with SCD [41, 42, 43, 44]. However, this strategy often eludes most children due to a lack of suitable donors, and many adults because of anticipated toxicities and age‐related organ dysfunction. Current nonmyeloablative (NMA) and reduced intensity (RIC) approaches, including related HLA‐haploidentical BMT with post‐transplantation cyclophosphamide (haplo‐BMT), offer the possibility to cure over 90% of eligible adults with severe SCD [52, 53].

### 2.3 Matched Sibling Donor AlloHCT

An HLA‐matched sibling donor (MSD) myeloablative alloHCT was first described over 40 years ago (in 1984) in a child with acute myeloid leukemia and SCD [54]. Since then, this approach has become the most common and arguably the most effective treatment for SCD. Currently, researchers are working to reduce toxicity without compromising its curative potential, which is preferred in adults with compromised organ function. AlloHCT using myeloablative conditioning with busulfan and cyclophosphamide was developed as a treatment for children and became the main approach in the late 1990s and early 2000s [41, 42, 43, 44]. Early studies showed promising results, with EFS rates of about 84%–86% and an OS rate of 93%–94% [41, 42, 43, 44]. Several improvements have increased EFS to 98% for patients under 30 years and achieved 100% survival in those under 5 years. It is now considered standard care at many centers worldwide. Myeloablative MSD alloHCT offers a high cure rate, with immunologic complications, such as severe GVHD, occurring in roughly 5% of patients. Additionally, fewer than 5% require long‐term systemic immune suppression, which remains a concern. Generally, the short‐term toxicities of MSD alloHCT are reversible; however, the morbidity associated with myeloablative conditioning can negatively affect patients' quality of life during the peri‐transplant period. Although rare, long‐term risks of myeloablative‐conditioning‐based alloHCT include an increased risk of secondary cancer, infertility, worsening of SCD‐related organ dysfunction, and metabolic problems [44, 50].

To reduce the toxicity associated with MSD alloHCT, several less intense regimens known as NMA or RIC have been developed [55, 56, 57, 58, 59]. These low‐toxicity regimens can achieve EFS and OS rates comparable to those of busulfan‐based myeloablative regimens, with stable mixed chimerism. AlloHCT, which results in stable mixed chimerism, has been shown to eliminate the SCD phenotype in adults [60, 61, 62]. However, the outcomes of this approach in reducing toxicity and achieving cures in children are still under evaluation [56]. Adults with SCD experience cumulative organ damage, making high‐intensity regimens unfeasible due to the risk of further organ injury during alloHCT. NMA and RIC regimens are characterized by lower toxicity, stable mixed chimerism, and autologous reconstitution if the transplant is complicated by graft failure, without needing a repeat donor stem cell infusion. This approach is preferred in adults with age‐related organ dysfunction [60, 61]. Some preliminary data also suggest that fertility may be preserved in female patients because this reduced toxicity regimen lowers alkylator exposure levels [58]. Unfortunately, suitable HLA‐matched sibling donors are only available for 8%–14% of individuals with SCD [41, 60, 61]. Another recent concern regarding NMA/RIC transplant methods is the risk of secondary myeloid neoplasms. AML/MDS has been observed following alloHCT for SCD, but only in host cells that were present due to either graft failure or mixed chimerism [46]. It is believed that the residual hematopoietic stem cells (HSCs) exposed to conditioning chemotherapy and radiation undergo significant regenerative stress when there is graft failure or low donor chimerism, resulting in clonal hematopoiesis [63]. The long‐term effects of these approaches still need to be studied, especially in the pediatric population [55].

Overall, the NMA and RIC approaches are more practical for LMICs, because they lead to less toxicity, fewer hospital stays, lower resource use, and fewer transplant‐related complications in areas where advanced supportive care is often unavailable. However, this must also be combined with the ability to monitor long‐term in settings where specialized care and late‐effects clinics are often missing. Table 2 outlines the various approaches for alloHCT in children and adults with SCD.

**TABLE 2:** Pros and cons of allo‐HCT as a curative therapy for individuals with SCD.

Allo‐HCT	Mechanism	Pros	Cons
General	Multiple (listed below)	Potential for cure Large cumulative experience Improving rates of engraftment More available to adults with SCD Evidence that successful transplant attenuates progressive vasculopathy and end‐organ damage Increasing donor availability Conceptually lower cost compared with other curative therapies	Long‐term‐ risk of ovarian failure, failure to achieve puberty, amenorrhea, secondary malignancies with non‐myeloablative/radiation‐based conditioning approaches Risk of clonal hematopoiesis of indeterminate potential (CHIP) before allo‐HCT in non‐engrafted patients or those with mixed chimerism Cost of specific approaches is prohibitive for LMIC Requires FACT‐accreditation Mostly available in HIC and a few LMIC
Matched sibling donor	Myeloablative (MAC) [41, 42, 43, 44]	Mainly in children 5‐year EFS 94%; OS 97% Graft rejection (1.4%) Mortality related to transplant (1.4%) Extended follow‐up: > 15 years	Limited donor availability (8%–14% of SCD patients) Graft rejection 7%–10%; TRM 7% Acute GvHD (20%) Chronic GvHD (10.5%) Late effects: acute lung injury, infertility, endocrine and metabolic issues Challenging in LMIC with lack of supportive care Available only in a few specialized centers in LMIC
	Non‐myeloablative/reduced intensity conditioning [55, 56, 57]	5‐year EFS 87%; OS 93% Extended follow‐up: > 15 years Minimal to no chronic GVHD Better tolerated in adults Possible fertility preservation Lower acute and chronic GvHD rates Mortality related to transplant (0%–5%) This approach is most applicable for LMIC, with low toxicity, low GVHD risk, lower cost, and scalability	Limited donor availability (8%–14% of SCD patients) Graft rejection 11%–14%; TRM 0%–5% Higher risk of graft failure (11%–14%) with NMA Optimal conditioning for adults remains unclear NMA > 50% with mixed chimerism, increasing risk of graft failure and myeloid malignancy risk Late effects: endocrine and metabolic issues, fertility may be preserved
Alternative donor transplantation			
Matched unrelated donor transplantation	Reduced intensity Regimen [64, 65, 66, 67]	HLA‐MUD transplant May increase donor pool (8/8 allele match) Median follow‐up was 26 months (12–62) Met prespecified 1‐year EFS, and significantly improved HRQL Unrelated cord blood transplantation (CBT) May increase donor pool (5–6/6 allele match) Median age 13.7 years (7.4–16.2 years) Median follow‐up of 1.8 years (1–2.6)	Limited donor availability EFS 69%; OS 79% Acute GVHD 28% Chronic GVHD 62%; 38%‐ extensive (12%‐CBT) Graft rejection (7%‐MUD; 63%‐CBT) Mortality related to transplant (24%) Complications: acute lung injury, PRES, intraventricular hemorrhage with seizures, infertility, EBV reactivation with PTLD—not considered safe Available only in a few specialized centers in LMIC
Haploidentical BMT with post‐transplant cyclophosphamide	Non‐myeloablative [68, 69, 70]	Most adults with organ dysfunction EFS 88%; OS 95% Reduced acute and chronic GvHD (10%) Expansion of regulatory T‐cells that promote immune tolerance Replicable at most transplant centers Low likelihood of developing EBV‐PTLD Low documented incidence of donor‐derived malignancies in the short‐term follow‐up. Most cost‐effective approach to develop in LMIC, which will expand the donor pool to most patients, with low toxicity, low GVHD risk, and scalable.	Graft rejection ~20%–25% in children Increased viral reactivation Increases risk of infertility secondary to additional alkylator therapy Short‐term follow‐up (< 10 years) Potential acute toxicity from high doses of cyclophosphamide, including cardiac, lung, bladder, and secondary malignancy from alkylating agent exposure Available only in a few specialized centers in LMIC
T‐cell depletion (TCD) T‐cell receptor alpha/beta^+^ and CD19^+^ depletion	Myeloablative [71] (MSD and Haplo) (NCT04201210)	Retain gamma/delta^+^ T‐cells (promote IR and provide pathogen‐specific immunity) and natural killer cells while depleting alloreactive T‐cells that cause acute GvHD Less delayed T‐cell‐specific IR EFS 88%; OS 95% No Grades III and IV acute GvHD	Requires even more specialized expertise than CD34^+^ selection methods of CD3^+^and CD19^+^ negative selection Available at fewer centers in HIC More data for children than adults 16% mild‐to‐moderate chronic GvHD Not cost‐effective in LMIC due to the need for a specialized facility

Abbreviations: Allo‐HCT, allogeneic hematopoietic stem cell transplant; EBV, Epstein–Barr virus; GvHD, graft‐versus‐host disease; HIC, high‐income countries; IR, immune reconstitution; LMIC, low‐ and middle‐income countries; MUD, matched unrelated donor marrow transplant; PTLD, post‐transplant lymphoproliferative disorder; TCD, T‐cell depletion; TCR, T‐cell replete.

### 2.4 Alternate Donor Transplant

The low likelihood of a patient with SCD having an MSD (estimated ~8%–14%) prompted the search for alternative donor options for alloHCT. Current methods to expand the donor pool for alloHCT in SCD include matched unrelated donors, umbilical cord blood, and related haploidentical donors. The use of unrelated donors with 8/8 matched HLA alleles has been disappointing [64]. To mitigate the increased risk of GVHD when using alternative donors, other agents, such as co‐stimulatory blockade and abatacept, are being studied as part of the GVHD prophylaxis regimen, with promising results [65]. Historically, graft failure has been a concern in unrelated cord blood transplants (UCB) for people with SCD, occurring in about 50% of cases [66]; however, recent strategies seem promising [67]. Exploring the use of unrelated volunteer donors or UCB transplantation is not a cost‐effective strategy for alloHCT in LMIC due to the lack of an ethnically diverse donor pool [72]. Most LMICs are not connected to accredited unrelated donor registries, and the substantial costs of developing, procuring, and maintaining UCB storage banks are quite prohibitive.

Related haploidentical BMT (haploBMT) with post‐transplant cyclophosphamide (PTCy) has become the standard alternative donor strategy when an MSD is unavailable, representing an evolution of the original Hopkins platform [52, 53]. This method expands the donor pool to over 90% of eligible individuals with SCD, decreases the risk of GVHD, and current strategies have lowered the graft failure rate linked to the initial approach [68, 69, 70]. HaploBMT using NMA/RIC has eliminated the toxicity barrier that previously limited its use in older adults with SCD and chronic end‐organ damage. Two recent Phase 2 multicenter studies of haploBMT with PTCy in adults with severe SCD (the BMT CTN 1507 and Vanderbilt's Global Haploidentical Learning Collaborative studies) [69, 70], with a combined total of 80 adult participants, showed a 2‐year EFS of 88% and 95%, and OS rates of 94% and 94%, respectively. The related haplo‐BMT approach, combined with thiotepa [69, 70] or low‐dose radiation (400 cGy TBI) [68], is a tolerated and scalable approach for increasing curative options for most eligible individuals with SCD and no MSD, even in LMICs. Another strategy employing T‐cell depletion (α/ß T‐cell) related haploidentical alloHCT has been employed in children and adults with SCD who lack sibling donors and have failed at least 1 year of hydroxyurea therapy [71]. While the results appear promising, scalability concerns for LMICs arise due to the need for specialized expertise and the increased toxicity of the myeloablative conditioning. Table 2 summarizes the advantages and disadvantages of the various alloHCT strategies as potential cures for SCD.

### 2.5 Unique Considerations for Using AlloHCT in SCD in LMICs

#### 2.5.1 Mortality Burden

The mortality burden of SCA is primarily concentrated in sub‐Saharan Africa. SCD mortality rates are highest among children, especially in countries with the highest under‐5 mortality rates [1]. Comprehensive strategies are crucial to address the morbidity and mortality associated with SCD, particularly in LMICs, where it significantly contributes to all‐cause mortality [1]. AlloHCT is a curative, scalable, and cost‐effective treatment option for eligible patients in sub‐Saharan Africa; however, it is currently only available in only seven countries across the continent. At present, the few patients with suitable sibling donors who can afford a transplant often have to travel abroad for the procedure and then return to their home countries, where there is a lack of expertise and resources for post‐transplant follow‐up.

#### 2.5.2 Infrastructure

The challenges of providing alloHCT in LMICs across Africa are significant. It is prohibitively expensive and requires specialized facilities that are not widely available in sub‐Saharan Africa. The healthcare infrastructure is underdeveloped, with a high prevalence of communicable diseases, a greater incidence of multidrug‐resistant pathogens, the lack of newer and more effective antimicrobial agents, and the unavailability of advanced transplant technologies such as T‐cell depletion, sophisticated HLA typing laboratories, and stem cell processing facilities. The absence of specialized supportive care, the high costs of new strategies, insufficient experienced staff, and the lack of chemotherapeutic and immunosuppressive agents further worsen the outlook [73, 74, 75, 76]. However, progress is being made in improving overall healthcare management and in addressing access to essential treatments and healthcare infrastructure. The HLA alleles in people of African descent are highly genetically diverse [76]. Finding a local or international unrelated donor or umbilical cord blood for a patient of African or mixed‐race descent is exceptionally challenging and may be prohibitively expensive [72, 76]. AlloHCT using related family donors, an MSD or a related haploidentical donor, is attractive as a platform that provides a readily available donor pool in LMICs, considering the increased fertility rate and larger family sizes of these populations, as well as limitations with cryopreservation. AlloHCT approaches using reduced‐dose conditioning (RIC/NMA) are also less toxic, and the simplicity of these approaches is noteworthy [60, 70].

#### 2.5.3 Capital Requirements

Most transplant centers in LMIC operate within regional tertiary facilities that are supported by government funding. Establishing partnerships among tertiary centers, government agencies, private or non‐governmental sectors is crucial for sustainability. International collaboration is essential to provide oversight, personnel training, and ancillary support for the program. Selecting highly effective transplant methods that have a low risk of complications like acute and chronic GVHD, and are affordable for patients and their families, is vital. In LMICs, improving the supply chain is critical, especially for access to cost‐effective antimicrobial resources [76], expanding the list of essential medicines as designated by the World Health Organization (WHO) [77, 78], or choosing drugs or strategies that prevent further complications and reduce out‐of‐pocket costs without sacrificing treatment quality. It is also vital to develop a long‐term follow‐up plan for recipients to monitor late effects after alloHCT, including chronic GVHD, graft failure, and fertility issues. Continuing to manage SCD‐associated comorbidities such as transfusion‐related iron overload, opioid use disorder, priapism, chronic leg ulcers, metabolic syndrome, and malnutrition is important. Malnutrition is widespread and likely one of the leading causes of death among children with SCA in Africa. It increases the risk of hospitalization and death [79]. Moreover, in some areas of Africa, HIV seroprevalence can be as high as 13% [80]. These factors must be addressed when considering alloHCT as a curative option. Table 3 outlines the minimum requirements for establishing an alloHCT program in an LMIC.

**TABLE 3:** Minimum requirements for developing an alloHCT program in a LMIC [73, 74, 75, 76, 77, 78, 79, 80].

	Description of needs	Inherent challenges	Potential solutions in LMIC
Local needs	**Institutional support for the following**: Cell Processing Laboratory Blood Banking and Transfusion Support HLA Compatibility and Chimerism Testing Therapeutic Drug Level Monitoring Microbiology Support, including Pathogen Drug Susceptibility Testing Radiology Services, Including x‐ray, CT, MRI, and Ultrasound Scans Surgical Capabilities and Support Placement of Central Lines Parenteral Nutrition Support and Expertise Pharmacy Support for Drug Access, ‐Including Chemotherapy Agents, Antimicrobials, and Immunosuppressive Agents Radiation Services for TBI Trained Personnel (Physicians, Nurses, Laboratory Staff) in HSCT and Ancillary Support. **Inpatient unit:** Clean, single‐occupancy rooms with isolation capability; HEPA‐filtered positive‐pressure rooms. **Outpatient clinic:** Exam rooms equipped for infusions **Intensive care unit**: access to vasopressors, dialysis, positive‐pressure, or mechanical ventilatory support	Few inadequate centers and funding sources Inaccurate data and a lack of guidance in needs assessment and feasibility reports Loss of income for patients, families, and caregivers Transportation costs and distance challenges for patients and families Limited access due to unfavorable socioeconomic conditions High drug costs, often unregulated. Lack of follow‐up care Insufficient availability and expertise in LMICs Brain drain of trained professionals in HSCT	Needs assessments by the transplant community, along with local hematologists Obtain accurate epidemiologic data with the assistance of governmental or non‐governmental organizations Seek professional consultations with management consulting firms if affordable Assess the use of generic drugs and biosimilars Train LMIC hematology physicians at a tertiary care hospital in HIC Secure institutional, governmental, and private commitment to support finances, quality control, ongoing training, personnel proficiency, and FACT accreditation Incorporate quality checklists for hospital management in the initial capital planning
Capital requirements	Total cost analysis for startup and ongoing sustainability expenses The number of inpatient beds is a key long‐term factor affecting both direct and indirect costs, mainly due to the specialized bed units needed for HSCT	Quality assessments and improvement programs are often not regularly included in initial capital expenses All aspects not initially considered (e.g., hospital engineering for positive pressure ventilated rooms, HLA typing facilities, optimal blood bank facility, etc.) Cost‐utility and cost–benefit analyses for most clinical management aspects are missing	Enhance health budget allocation as recommended by the WHO Include quality checklists for hospital management in the initial capital Clearly define start‐up HSCT program priorities with input from multiple experts both within and outside the institution (e.g., donor sources, conditioning, medications, etc.) Increase healthcare workers' salaries to help reduce the brain drain of trained professional personnel
Leadership and expertise	A medical director who is a licensed hematologist with at least 1 year of training in a BMT unit Applies to physicians, nurses, technicians, and all other allied health staff	Lack of HSCT expertise—For starting a new alloHCT program, including experienced and well‐trained transplant physicians and nurses	Focus on human resources to recruit enough well‐trained and experienced staff with expertise in alloHCT^a^ This includes nursing, physician, and laboratory personnel. International collaboration is required
Quality and sustainability	The quality of the program encompasses many aspects: Quality of patient care (including safety) Laboratory quality control procedures (3) Value determinants serving as proxies for program quality, such as clinical outcomes and patient‐reported outcomes	Maintaining the quality of an alloHCT program requires additional staffing resources. Information technology tools, error monitoring, and cost‐control measures that may increase expenses.	For the sustainability of a new alloHCT program, it is essential to convince leadership, entrepreneurs (and/or venture capitalists), and government authorities that focusing on quality is vital in alloHCT, given the high stakes involved in this tertiary care service Follow WBMT recommendations for establishing an HSCT unit at three levels of care: minimum, preferred, and ideal Develop functional and practical health insurance systems Utilize “twinning” or “pairing” strategies for ongoing advice over the years (e.g., 5‐year contract/Memorandum of Understanding) with experienced alloHCT programs in other countries Ideally, automate compliance measurement tools to avoid errors, which can result in substantial cost savings
Accreditation	The goal is to establish international standards for certification by organizations related to HSCT. At least two accreditation bodies should have a necessary relationship with HSCT programs: Stem cell standards and Blood bank standards	Setting up a new program in a resource‐limited country to meet all the standards for an HSCT program in an HIC is costly	LMIC HSCT programs should report transplant activities to both local and global registries Both FACT‐JACIE and blood bank accreditation organizations (e.g., AABB) have basic and optimal standards for accreditation The goal should be to obtain certification within a few years of the first transplant. Getting direct advice from FACT or JACIE is essential for enhancing standard policies and procedures

Abbreviations: AABB, American Association of Blood Banking; CT, computed tomography; FACT, Foundation for the Accreditation of Cellular Therapy; HEPA, high efficiency particulate air filter; HIC, high‐income countries; HSCT, hematopoietic stem cell transplant; JACIE, Joint Accreditation Committee—ISCT and EBMT; LMIC, low‐middle‐income country; MRI, magnetic resonance imaging; PROM, patient‐reported outcome measures; US, ultrasonography; WBMT, Worldwide Network for Blood and Marrow Transplantation.

^a^ The exact needs for an allogeneic hematopoietic stem cell transplant (alloHCT) should be assessed using both demographic and economic analysis, which are significantly challenging in LMICs.

#### 2.5.4 Patient Reported Outcomes

Patients with SCD are known to have a lower HRQL [10, 11, 12]. In addition to the physical symptoms of SCD, psychological and social stressors, as well as academic struggles, often affect patients due to the chronic nature of the disease and its complications [50]. In a patient‐reported analysis using adjusted mixed‐effects models, patients showed significant improvement in most aspects of HRQL within 1 year after alloHCT, in addition to alleviating the clinical manifestations of SCD. Similar results were observed across the physical, social, and emotional domains of HRQL after adjusting for demographic and medical variables [51]. HRQL is typically assessed using validated questionnaires, such as the CHQ and the PROMIS, a set of person‐centered measures that evaluate and monitor physical, mental, and social health in adults and children before and after transplant [49, 50, 51]. Most commonly used generic HRQL measures were developed in Europe or North America. They may not be appropriate for use in LMICs, where cultural differences are more pronounced compared to those in the West. Health is a concept deeply rooted in culture, and due to these differences, it cannot be assumed that a well‐established Western measure of HRQL is always suitable in other cultural settings. Careful evaluation and testing of conceptual equivalence are essential when determining whether to use an existing HRQoL measure outside the culture in which it was developed.

#### 2.5.5 Risk of Malignancy

Although the relative risk of hematologic malignancies is higher in SCD, the absolute risk remains low [81, 82]. Lawal et al. found that the rate of hematologic malignancy was approximately 45 times higher following alloHCT for SCD in adults with graft failure and mixed chimerism compared to those with SCD who did not undergo curative therapy. This finding aligns with reports following gene therapy [46]. The true incidence of post‐transplant cancer after current curative treatment for SCD remains unkown, and long‐term prospective data will be required. Monitoring this through a coordinated registry with the institution of alloHCT in LMICs will be essential. Consequently, conditioning that promotes complete donor engraftment and reduces the risk of secondary myeloid neoplasm is probably preferred.

## 3 Gene Therapy Using Autologous HCT as a Potential Cure for SCD

### 3.1 Background

Gene therapy and editing for SCD have advanced to clinical use with (i) a lentiviral vector technology delivering an anti‐sickling “fetal‐like” β‐globin gene (bluebird bio) and (ii) CRISPR technology to knock out Bcl11a, the repressor of fetal hemoglobin, in an erythroid‐specific manner (CRISPR/Vertex). Both methods have shown significant increases in antisickling globin, reversing SCD symptoms. However, both come with a hefty price tag of $2–3 million per patient, making them unaffordable for healthcare systems and unimaginable in LMICs. In fact, because the European healthcare system did not endorse covering the costs of genetic therapy, bluebird bio pulled the product from the European market despite EMEA approval. More recently, after receiving FDA approval in December 2023, bluebird bio was acquired by a private equity group at a relatively low cost.

Genetic therapy must be both affordable and accessible to everyone. Achieving this requires a coordinated effort to remove barriers related to technology transfer and make gene therapy cost‐effective. This starts with grassroots initiatives, including raising awareness and providing education within affected communities, as well as among regulatory agencies and governments, building the basic infrastructure needed for delivering, testing, and monitoring treated patients is crucial. Addressing the technical challenges will help lower complexity and costs, as well as improve access to gene therapy, are described below.

Gene therapy entails collecting HSCs, purifying them from non‐stem cells, performing genetic manipulation, administering chemotherapy conditioning to the patient, and infusing the genetically manipulated cells. The section below discusses the barriers that need to be overcome to bring genetic therapies to LMIC, especially in sub‐Saharan Africa.

- Improving HSC Collection

Obtaining sufficient hematopoietic stem and progenitor cells (HSPCs) for genetic manipulation is challenging in SCD, as nearly 5–10 times the CD34^+^ cell dose is required for gene therapy [83, 84, 85, 86] due to (a) HSPC losses during ex vivo selection and genetic manipulation, (b) safety testing of the gene‐modified product, (c) back‐up HSPC cryopreservation with myeloablative conditioning, and (d) loss of “stemness” during culture and genetic manipulation [87]. The two currently FDA‐approved gene therapies [88, 89] require 10 times more HSC collection than in alloHCT [90, 91].

Large HSC cell dose requires multiple rounds of HSC mobilization and collection, mainly because (i) G‐CSF, which is known to mobilize HSCs into the blood in large numbers, is contraindicated in SCD [92]; (ii) the bone marrow microenvironment is compromised by inflammation, and altered vasculature [93, 94]. (iii) Hydroxyurea, used to induce fetal hemoglobin, is a cytotoxic drug that affects CD34^+^ cell yield [94]. Hence, 5%–10% of patients where this HSPC collection dose is not met become ineligible for gene therapy. Bone marrow harvests have shown poor yield and recovery in the initial patients treated [95], because the yield and recovery are poor, and there are fewer primitive HSCs in SCD bone marrow [94]. Hence, all current lentiviral gene therapy and gene editing trials for SCD rely on multiple cycles of plerixafor followed by apheresis.

Multiple HSC collections increase the burden on patients, requiring more placement of more permanent apheresis catheters, pre‐collection erythrocytopheresis to reduce HbS levels, multiple rounds of CD34^+^ cell purification, and cryopreservation. These steps raise overall costs and significantly delay gene therapy infusions (each cycle of apheresis can only be repeated 2 weeks apart). Additionally, a large dose of HSPC cells demands a high amount of expensive, clinical‐grade vector or gene editing reagents.

*Strategies to reduce the complexity and costs of HSPC collections*:

- Discontinuation of hydroxyurea and regular blood transfusions for 3 or more months to significantly improve the quantity, quality, and yield of HSPCs collected [93, 94, 95]
- Optimizing mobilization to a single 1‐day collection. A higher dose of plerixafor has been shown to increase CD34^+^ cell mobilization in patients with SCD [96]. A newer CXCR4 inhibitor peptide, Motixafortide, more potent at mobilizing HSPCs, was approved by the FDA in 2023. Adding a second mobilizer like Groβ (MGT‐145, a CXCR2 agonist), Meloxicam (an NSAID), or propranolol (a beta‐2 adrenergic blocker) in combination with Plerixafor or Motixafortide. Combinations of mobilizers can improve HSC yield, reducing the number of apheresis procedures required.
- Optimizing the technicalities of the apheresis procedure, which includes timing of apheresis after mobilizing agent [97], collect deeper into the buffy coat, increasing the anticoagulant ratio, altering apheresis centrifugation parameters, and performing apheresis after HbS reduction (< 30%), with erythrocytopheresis or chronic blood transfusions, improves stem cell mobilization and recovery [98, 99, 100].

II HSC Enrichment of the Apheresis Product

An apheresis cell product consists of mononuclear cells (MNCs), which contain mobilized CD34^+^ cells. All gene therapy approaches for hematopoietic disorders, including SCD, target CD34^+^ HSPCs using a closed magnetic bead isolation system, the CliniMACS or Prodigy. The CD34^+^ HSPCs are a heterogeneous mix of mostly progenitor cells, with only 1%–2% HSCs possessing long‐term multilineage engraftment potential [101]. As a consequence, current gene therapy approaches targeting CD34^+^ HSPCs are aimed chiefly at progenitor populations, which are short‐lived, and are not efficient at targeting the rare long‐term repopulating HSC. The phenotype of HSCs is CD34^+^CD38^−^CD90^+^CD45RA^−^CD49f^+^ [102]. If a more HSC‐enriched subset is isolated and targeted for gene therapy, it can (1) significantly reduce the number of modifying reagents needed for gene therapy and (2) result in more reliable genetic modification of HSCs, making gene therapy a success predictably.

*Strategies for enriching HSCs*:

- Enriching CD34^+^ cells one step further by purifying CD34^+^ CD38 low/− [103, 104] or CD34^+^ CD133^+^ [105] or CD34^+^ CD90^+^ [106, 107] can significantly enrich the HSC population (by reducing manipulated cells by approximately 10‐fold), can be clinically feasible, and provide long‐term repopulating potential in xenografts and non‐human model primates [105]. This will allow the same amount of gene therapy reagents to treat 10 patients.
- Recently, Radtke et al. compared the isolation of CD34^+^CD38^−^ and CD34^+^CD133^+^ cells to the isolation of CD34^+^CD90^+^ cells and showed a 30‐fold enrichment of CD34^+^CD90^+^ HSCs without compromising the engraftment potential in vivo, with approximately three times higher genetic modification of engrafted gene‐modified cells using this approach.

III Improving Lentiviral Vectors

Lentiviral vectors used for treating SCD comprise adding an antisickling fetal/fetal‐like globin gene (either a mutant β‐globin, β^T87Q^‐globin, or modified γ‐globin [γ^G16D^‐globin]) [88, 93], inducing endogenous γ‐globin gene expression by knocking down its repressor, Bcl11a, in an erythroid‐specific manner [105]. Gene editing approaches also utilize the strategy of activating fetal hemoglobin by reducing Bcl11a expression. The focus is on lentiviral vectors, where technological advances have occurred over the last three decades. Improving gene editing tools is not reviewed herein, as the field is rapidly evolving. High lentiviral vector titers (transducing vector particles/mL) reduce the amount of vector required for HSC manipulation.

*Strategies to improve lentiviral vectors*:

- Reducing vector size: The vector delivering a shRNA to Bcl11a is smaller and therefore has higher titers. Vectors delivering the globin genes have modest titers, as a large locus control region (LCR) enhancer is necessary for high‐level expression of the globin genes. Large inserts result in incomplete vector RNA genomes and lower viral titers [107]. Several attempts have been made to shorten the LCR or the γ‐globin gene [108, 109], but at the cost of reduced globin gene expression [109], unless combined with shRNA to Bcl11a and ZNF410 to activate endogenous γ‐globin [110].
- Modifications in the packaging of lentiviral vectors can also improve titers [111]. Production in bioreactors with suspension packaging cell lines, specific advances in transfection formulation methodologies, and genetic alterations of the packaging cells can result in high‐titer vectors at large scale. A large batch can help substantially reduce vector manufacturing costs [111, 112, 113, 114].

IV Improving Gene Transfer Into HSC and Closed‐System Processing

For ex vivo gene therapy to be accessible in LMIC, performing gene transfer in a closed‐processing system that eliminates the need for Clean Rooms and GMP processing, using systems that are transportable to provide point‐of‐care manufacturing of the apheresis product, is necessary. The Prodigy has been adapted for T‐cell selection of an unmobilized MNC apheresis product, followed by its transduction/editing for CAR‐T‐cell generation [115], and is a paradigm for developing an affordable, genetically manipulated HSPC production [116]. Here, the vector and MNC apheresis product are attached to the Prodigy, and the equipment selects the T‐cells, transduces them with the CAR‐lentivirus vector, and provides a closed bag of the transduced CAR‐T cells, which can be directly infused into the patient.

*Strategies for improving gene transfer and developing closed‐system HSC gene transfer*:

- A similar strategy for isolating an HSC‐enriched population from mobilized peripheral blood apheresis product and its modification with lentivirus on the Progidy is a practical approach that can be possible in LMIC.
- Closed‐system magnetic sorting of HSC‐enriched cell population: Masiuk et al. developed a dual magnetic sorting for CD34^+^CD38^−^ cells with a 7‐ to 10‐fold enrichment of HSCs, which can be adapted to a closed system processing in equipment like the Prodigy. Developing a closed system for processing CD34^+^CD90^+^ cells using novel systems can further enrich them by ~30‐fold.
- Improving gene transfer into HSC‐enriched populations via small molecules, such as UM171 [117] and SR1 [118], along with transduction enhancers, such as poloxamer F108, cyclosporin H, and prostaglandin E2, can significantly enhance HSC gene transfer [119, 120]. Many approaches to improving gene transfer while preserving “stemness” are under investigation and are beyond the scope of this article. The reader is referred to several recent reviews and advances [121, 122, 123, 124, 125, 126]

V HCT Conditioning Delivered on an Outpatient Basis

Two FDA‐approved gene therapies for SCD [88, 89, 95], and recent lentiviral gene therapy and gene editing trials for SCD in the pipeline [88, 89, 127], utilize myeloablative doses of busulfan. Myeloablative conditioning results in significant toxicity with a median hospital stay of 35 days, median times to platelet and neutrophil engraftment of ~35 and 20–27 days, respectively [88, 89]. These increase cost and complexity: requiring central venous access, significant blood product and infection support, and parenteral nutrition, and can only be done at well‐equipped tertiary care transplant centers. For LMICs, conditioning, when performed in an outpatient setting, can significantly reduce costs and complexity, as well as minimize patient toxicity.

*Strategies to reduce conditioning intensity, allowing outpatient gene therapy*:

- Our group has used reduced intensity conditioning with melphalan and observed median duration of 5 and 8 days of thrombocytopenia and neutropenia, respectively [128], making it feasible to be performed on an outpatient basis in the foreseeable future. This dose is administered to patients with multiple myeloma, who are mostly elderly.
- Antibody‐based conditioning is another less toxic alternative approach to reduce the adverse effects of chemotherapy conditioning and has the potential for outpatient gene therapy. To this end, an anti‐kit antibody targeting and depleting c‐KIT^+^ cells, the stem cell factor receptor present on HSPCs in the recipient's bone marrow, was tested in a Phase I/II clinical trial (NCT05357482). However, it was withdrawn for HSC gene therapies without reasons being disclosed. Another targeted antigen is CD45, a pan‐leukocyte surface protein. Proof‐of‐concept studies using an anti‐CD45 PBD antibody–drug conjugate, where the antibody is linked to a cytotoxic drug, have shown promising results in NSG mice [129]. Recently, Larochelle and colleagues have demonstrated that targeting Mpl with an antibody is another way of depleting recipient HSCs [130]. Antibody‐based approaches will significantly reduce the chemotherapy‐related toxicities and be applicable in LMICs.
- In vivo *gene therapy*: Recently, in vivo gene editing using lipid nanoparticles has been successfully used to treat an otherwise fatal genetic disorder [131]. If HSCs can be efficiently directly targeted in vivo, that would be ideal and most cost‐effective “off‐the‐shelf” gene therapy. The future of gene therapy in LMIC, and especially in sub‐Saharan Africa, lies in an in vivo delivery. Efforts are currently ongoing to translate this to patients.

*The current status of in vivo gene therapy*:

Currently, two major approaches for in vivo gene therapy of HSC are undergoing preclinical studies to be translated into clinical trials for patients.

- Helper‐dependent Adenovirus (HdAd) vectors, which can carry a huge cargo, do not integrate into the genome and can be delivered in vivo. HdAd has been used to deliver a variety of “cargo” to transfer or edit genes [132, 133]. Lieber et al. delivered CRISPR base editors and prime editors to correct SCD in mice [132, 133]. Here, HdAd carries the CRISPR sgRNA/pegRNA and Cas9 nuclease fusion to either adenosine deaminase (A → G base editor), which converts the HbS mutation to HbG‐Makassar using reverse transcriptase (prime editor), to correct the HbS mutation. They also carry the MGMTP140K gene as a drug selectable marker in the construct, which will select edited HSC by administering BCNU, a nitrogen mustard chemotherapy, to patients. HSPCs are mobilized in peripheral blood to target them with the IV‐injected adenovirus. The percentage of HSCs modified using this approach is low and, therefore, requires in vivo selection with BCNU. This approach is elegant but requires HSC mobilization and chemotherapy to induce replication of the few modified HSC, subjecting them to tremendous regenerative stress. They have successfully treated SCD mice and demonstrated proof of concept in non‐human primates and immune‐deficient mice engrafted with human cells using this approach and are poised to take it to the clinic [132, 133, 134].
- Lipid nanoparticles (LNP) to deliver CRISPR RNAs or RNA‐gene writers [135, 136]. Breda et al. have tested cKIT/LNPs carrying luciferase, Cre‐recombinase, PUMA, a proapoptotic gene, or an adenine base editor to convert the SCD mutation to Hb‐Makassar [124]. Although they observed extremely high delivery of Cre‐recombinase into Td‐tomato reporter mice (55% HSC marking after a single IV injection), and 58% loss of HSC with delivery of PUMA, the base editor for correcting SCD was only demonstrated in vitro. Nevertheless, proof‐of‐concept studies of in vivo delivery show promise, can be refined, and perhaps eventually become translatable.

If these in vivo approaches are successful, they would represent paradigm‐changing methods for genetically modifying HSCs.

## 4 Practical Considerations for Curative Therapies in Africa

The current curative therapies for SCD seem to offer lasting benefits. They could be transformative for most individual recipients; however, there are many additional considerations to be made in discerning the feasibility and implementation of these interventions in LMICs. From many perspectives, choosing between different forms of curative therapies and effective SCD management is not necessarily a binary decision. However, in situations of significant resource scarcity, pursuing high‐impact, high‐cost interventions for only a small few could hinder some countries' ability to adopt and expand SCD screening, diagnosis, and the use of less expensive, potentially life‐saving interventions for a larger portion of individuals with SCD, such as vaccinations, hydroxyurea, and malaria treatment. Today, there are very few examples of government support for national or regional SCD diagnosis and treatment programs in LMICs, despite some enthusiastic endorsements for establishing alloHCT centers and initiating gene therapy. While advances in curative therapies do not necessarily conflict with public health policy, pursuing both may present ethical and financial challenges for governments and non‐governmental funding agencies aiming to improve SCD outcomes in LMICs [137, 138].

Recommendations within the National Academy of Medicine (NASEM) 2020 Blueprint (https://www.nationalacademies.org/our‐work/addressing‐sickle‐cell‐disease‐a‐strategic‐plan‐and‐blueprint‐for‐action) and the Lancet 2023 Commission (https://www.thelancet.com/commissions/sickle‐cell‐disease) reports address stem cell therapies for SCD [139]. The Lancet Commission, which focuses heavily on lower‐resourced countries with a high SCD burden, recommends the accelerated development of these stem cell therapies to provide safe and accessible cures globally by 2040. It also suggests that future clinical trials of curative approaches should include patients from both HICs and LIMCs. Challenges in SCD, as outlined in the NASEM report, and strategies to address them focus heavily on the United States. However, when discussing curative therapies, they note barriers such as lack of knowledge, the availability of donors, and relationships with caregivers that may be universal. Knowledge about alloHCT for SCD is low. Accessible, unbiased educational tools are needed to increase literacy. The report advocates promoting more widely available approaches to SCD that do not require highly specialized institutions and expertise. It encourages the development of in vivo gene therapy, which will hopefully be a less demanding technology to implement.

### 4.1 Reproductive Health

Data on fertility outcomes after curative therapy for SCD are limited. Fertility in SCD is probably affected by pre‐transplant ovarian reserve or semen analysis results, which may already be abnormal due to SCD‐related damage, hydroxyurea treatment, and conditioning regimen. SCD may be inherently associated with reduced reproductive capacity for both men and women [140, 141]. Myeloablative chemotherapy, which is a requisite component of most current curative strategies, carries a risk of infertility, irrespective of the underlying condition for which transplantation is being pursued [142, 143]. Myeloablative conditioning causes severe gonad toxicity, especially among post‐pubertal females [142]. RIC and NMA conditioning might be less harmful to gonads in the short term; however, more long‐term fertility data are needed for these methods [144, 145]. Other factors that affect outcomes include differences in pubertal stage at alloHCT, follow‐up durations, the lack of pre‐transplant information, variations in reported results, and the absence of data on pregnancy attempts and/or infertility diagnoses [44, 142, 143, 144, 145].

Fertility preservation programs are limited in LMICs, and ongoing post‐transplant fertility care is necessary to address this gap. This creates an additional personal burden for individuals with SCD who wish to have children, and on families who are making critical decisions in the best interest of their underage children who may be too young to articulate a parenting plan. Fertility preservation is not universally accessible, and the costs of some options vary significantly due to procedural complexities, the probable need for long‐term storage of cryopreserved specimens, and the more experimental, limited approaches available for pre‐pubertal children. Without resources and coverage for these fertility preservation services, and subsequent assisted reproduction procedures in LMICs, patients with SCD and their families may face unavoidable compromises until advances in the field, such as non‐genotoxic, antibody‐mediated stem cell depletion, reduce the risk to future fertility.

### 4.2 Patient Selection

The current indications for transplantation differ depending on the curative approach, with broader criteria for alloHCT using stem cells from matched sibling donors, including stroke, elevated TCD velocities, recurrent acute chest syndrome, priapism, and pain. So far, the indications are relatively limited for genomic therapies, where clinical trial endpoints mainly focus on pain events requiring opioids and repeated acute care visits. There is little experience with gene therapy for other serious SCD manifestations.

Exclusion criteria for transplantation often include reduced lung, kidney, and heart function, citing increased risk of alloHCT‐related complications and death using myeloablative conditioning. Existing heart, lung, kidney, liver, and brain injuries in SCD, such as Moya‐Moya and severe CNS vasculopathy, may create additional barriers that limit access regardless of location. The ability to detect, effectively manage, or ideally prevent SCD complications may be limited in LMICs, which could further restrict access to or raise risks of alloHCT.

### 4.3 Geographic Limitations

AlloHCT is available in only six of the 54 African countries: Algeria, Egypt, Morocco, Nigeria, South Africa, and Tunisia [146]. du Toit et al. demonstrated the feasibility of haploidentical HCT, where a younger parent or offspring donor can be a viable option for adults in sub‐Saharan Africa with hematological malignancies who lack a suitable related or unrelated donor [147]. The Worldwide Network for Blood and Marrow Transplantation (WBMT), in collaboration with the African Blood and Marrow Transplantation (AfBMT), is exploring international support and collaboration to provide expertise tailored to local resources and regional population needs. Local engagement involving both government and private participants is essential to initiate and develop local alloHCT capabilities [146]. Establishing local alloHCT capability is crucial for improving overall health outcomes in affected countries. Several partnerships are exploring gene therapy and gene editing in Africa; however, implementation is still in progress, and significant challenges remain to be overcome [148].

### 4.4 Other Critical Infrastructure Challenges

Investments in some broader components of medical systems within LMICs are necessary to ensure the safety and feasibility of curative therapies. Health authorities must approve a list of essential medications, and facilities need to have ready access to agents for preparative conditioning, management of common acute toxicities, and handling of anticipated complications such as GVHD, including immunosuppression therapy.

The risk of infection with all HSCT approaches can be reduced to some extent. However, laboratory capacity to identify bacterial, viral, and fungal pathogens, along with access to appropriate treatments for infections, is crucial. Additionally, providing protective environments within patient care facilities can further decrease infection risk.

Currently, all forms of curative therapies for SCD will inevitably require blood products. Although some LMICs have national blood services, the demand for blood products from these procedures could easily exceed the supply. Access to platelets for transfusions is expected; however, the safe collection and storage of platelets pose challenges in the HSCT setting.

### 4.5 Advocacy and Awareness

The education and engagement of a global SCD community, which is geographically dispersed and may have varying levels of health literacy, will be additional considerations for achieving equity. Surveys of SCD patients in higher‐resource countries continue to demonstrate significant knowledge gaps regarding procedures used in curative therapies, the scientific basis for newer genomic therapies, risks, and outcomes [149, 150, 151]. These deficits are likely the same or greater in LMICs. Preparation and widespread distribution of multimedia materials across multiple platforms, offered in numerous languages, will be essential. Education of healthcare providers worldwide on these newer treatment options is still needed to support patients and their families in making informed decisions.

### 4.6 Technology and Regulatory Requirements

Technological advances may address challenges in curative therapy by expanding the potential donor pool and reducing the associated toxicity. Autologous curative approaches are maturing and will likely play a crucial role in the future, especially for individuals with SCD seeking curative options who do not have related family donors [41, 57, 68, 69, 70]. Current methods using related haplo‐BMT with PTCy have significantly increased the donor pool for alloHCT [68, 69, 70]. Most approaches in late‐stage clinical trials or those recently approved for commercial use by regulatory agencies in higher‐resource countries have demonstrated acceptable safety and efficacy. However, the regulatory infrastructure in many LMICs is less established and less resourced, which could lead to increased risks and reduced effectiveness. While ex vivo gene therapy is emerging as an alternative option, in vivo gene therapy may become a more affordable and accessible treatment in the future. With the potential to cure many more patients soon, substantial resources must be allocated worldwide to achieve this.

### 4.7 Economic Barriers

Several disease‐modifying therapies are currently available for SCD, including hydroxyurea, antimetabolites, and blood transfusions, which are accepted as the standard of care [73]. Several new therapies, including crizanlizumab (a monoclonal antibody), voxelotor (a small molecule), and l‐glutamine (a naturally occurring amino acid), have recently been approved by the FDA [13]. Curative therapies, including alloHCT and autologous gene therapy, come with a substantial cost and are associated with a wide range of complications. Conducting a cost‐effective analysis (CEA) of these therapies to accurately include all significant costs and outcomes can be challenging, especially in LMICs, where fewer CEA studies have been done and results may differ across regions.

In high‐income countries (HICs), the lifetime burden of total medical costs attributable to SCD for non‐elderly individuals is estimated at $1.7 million. In comparison, patients incur approximately $44 000 in out‐of‐pocket expenses over their non‐elderly lives, with no differences observed between sexes [152]. The median total healthcare cost for patients undergoing myeloablative alloHCT in HICs is estimated between $289,283 compared to $253, 467 for those receiving non‐myeloablative or reduced‐intensity alloHCT [153]. AlloHCT is offered at a lower cost in some LMICs, typically by using more affordable methods. However, long‐term data are needed to evaluate the outcomes of these approaches over time [154, 155].

Currently approved gene therapy options have a price tag of $2 to $3 million. Although they can significantly improve both life expectancy and quality of life, the cost is prohibitive even in HICs. Comparative modeling analysis using two independently developed simulation models indicates that a gene therapy for SCD, priced below $2 million, is likely to be cost‐effective when assessed from a locally derived societal perspective and an equity‐focused CEA threshold [156, 157].

## 5 Conclusions

SCD is becoming increasingly important to global public health due to its high contribution to all‐cause mortality in LMICs, which might not be evident when each death is attributed to a single cause [1]. A comprehensive care strategy to reduce the related morbidity and mortality in low‐resource settings should include both comprehensive care programs and curative therapies. AlloHCT is currently the most cost‐effective curative therapy option for addressing this global disease burden. Advancements in transplant technology and supportive care have made this approach adaptable worldwide [60, 70]. NMA and RIC regimens with lower intensity have expanded alloHCT to older patients with SCD and comorbidities [57, 58, 59, 60, 61, 68, 69, 70]. These non‐toxic conditioning regimens, easily administered in the outpatient settings, could significantly increase the use of alloHCT in LMICs and are estimated to be nearly 50% cheaper [154, 155]. Current curative treatments for SCD in clinical practice and research involve complex procedures and facilities that are often inaccessible in many LMICs, where most of the global sickle cell population resides. However, several LMICs, such as Bangladesh, South Africa, and Tanzania, have performed an increasing number of alloHCT procedures using locally adapted approaches [74, 75, 76]. Fostering collaborations among academic institutions, private industry, and governments should be a priority. Key considerations include focusing on technologies that enable single infusions with long‐lasting effects, supporting early detection and evidence‐based early interventions within local healthcare systems, and developing financial and workflow models to broaden access to curative treatments [156]. The current estimated cost for gene therapy is prohibitive [157]. To expand the use of gene therapy in SCD, several key areas need to be addressed: the collection and conditioning of stem cells, long‐term data demonstrating success, improvements in stem cell collection and conditioning protocols, increased expertise, and cost reductions. Achieving these goals will likely take decades. Other patient factors may also limit the use of either therapy. While expertise is essential, considering the extensive experience in the allogeneic setting, alloHCT might be more practical for wider adoption in the short term. There is a need to accelerate the development of effective and affordable curative therapies, including prioritizing scientific and infrastructure investments to ensure safe and accessible cures globally. This requires action from governments, pharmaceutical companies, and research funding organizations. We must ensure a balanced participation of LMICs and HICs in clinical trials for new therapies by 2030, involving drug licensing authorities, pharmaceutical companies, research funding bodies, and researchers.

## Author Contributions

A.A.K., P.M., and A.A.T. wrote and edited the manuscript. All authors read and approved the final manuscript and accept accountability for all aspects of this work.

## Ethics Statement

Approval from the ethics committee is not required. The manuscript is a review of the current literature on the topic and does not report results of original experimental investigations conducted by the authors. The authors' institutional review boards have approved it.

## Conflicts of Interest

The authors declare no conflicts of interest.

## Data Availability

The data supporting the findings of this study are available from the corresponding author upon reasonable request.
